# Direct observation of ultrafast plasmonic hot electron transfer in the strong coupling regime

**DOI:** 10.1038/s41377-019-0121-6

**Published:** 2019-01-16

**Authors:** Hangyong Shan, Ying Yu, Xingli Wang, Yang Luo, Shuai Zu, Bowen Du, Tianyang Han, Bowen Li, Yu Li, Jiarui Wu, Feng Lin, Kebin Shi, Beng Kang Tay, Zheng Liu, Xing Zhu, Zheyu Fang

**Affiliations:** 1 School of Physics, State Key Lab for Mesoscopic Physics; Academy for Advanced Interdisciplinary Studies; Collaborative Innovation Center of Quantum Matter, Peking University, 100871 Beijing, China; 2 CNRS International-NTU-Thales Research Alliance (CINTRA), Nanyang Technological University, Singapore, 637553 Singapore; 3 Centre for Micro-/Nano-Electronics (NOVITAS), School of Electrical and Electronic Engineering; Centre for Programmed Materials, School of Materials Science and Engineering, Nanyang Technological University, Singapore, 637553 Singapore

**Keywords:** Nanophotonics and plasmonics, Ultrafast photonics

## Abstract

Achieving strong coupling between plasmonic oscillators can significantly modulate their intrinsic optical properties. Here, we report the direct observation of ultrafast plasmonic hot electron transfer from an Au grating array to an MoS_2_ monolayer in the strong coupling regime between localized surface plasmons (LSPs) and surface plasmon polaritons (SPPs). By means of femtosecond pump-probe spectroscopy, the measured hot electron transfer time is approximately 40 fs with a maximum external quantum yield of 1.65%. Our results suggest that strong coupling between LSPs and SPPs has synergetic effects on the generation of plasmonic hot carriers, where SPPs with a unique nonradiative feature can act as an ‘energy recycle bin’ to reuse the radiative energy of LSPs and contribute to hot carrier generation. Coherent energy exchange between plasmonic modes in the strong coupling regime can further enhance the vertical electric field and promote the transfer of hot electrons between the Au grating and the MoS_2_ monolayer. Our proposed plasmonic strong coupling configuration overcomes the challenge associated with utilizing hot carriers and is instructive in terms of improving the performance of plasmonic opto-electronic devices.

## Surface plasmons: Nanostructured sandwich stirs up hot electrons

A device that taps into two types of surface plasmon waves offers new opportunities to transform light energy into speedy semiconductor charges. When surface plasmons are confined to nanoscale dimensions, they can rapidly decay and produce “hot” electrons with high kinetic energy. Zheyu Fang from Peking University in Beijing, China, and colleagues have constructed a metal–insulator–metal sandwich structure that improves harvesting of hot electrons for applications including photodetectors. To increase extraction under laser excitation, the team coated the insulator with an electron-attracting molybdenum disulfide monolayer. They also identified surface plasmon polaritons—mobile waves at the metal–insulator interface—in the sandwich structure using a gold grating with variable dimensions as the upper metal layer. Laser conditions that coupled mobile and localized plasmons promoted hot electron production instead of thermal dissipation.

## Introduction

Surface plasmons (SPs), as the collective oscillation of free electrons at the interface between dielectric and metal layers^1^, have aroused tremendous interest in diverse fields, such as solar energy conversion, superresolution, high harmonic generation, near-field imaging, and nonlinear phenomena^2–9^. As nonpropagating SPs, localized surface plasmons (LSPs) can either dephase radiatively by re-emitting photons or decay by Landau damping to form energetic electron−hole pairs^10,11^. These pairs are nonthermal, and their intense collisions can redistribute accumulated energy in hundreds of femtoseconds, developing into hot carriers that obey a Fermi−Dirac-like distribution with an increased effective temperature^2,3^. If these hot carriers are exported at a rate faster than energy dissipation by electron−phonon scattering, they can be collected and utilized in external circuits for opto-electronic devices such as photodetectors^12–26^. To realize this application, two critical challenges must be overcome: the large radiative rate of LSPs and the rapid relaxation of the hot carriers.

In contrast to LSPs, surface plasmon polaritons (SPPs) relax almost nonradiatively even at rough surfaces, leading to a higher photon-to-carrier conversion efficiency^27,28^. However, such carriers with lower energies have a low probability of crossing the potential barrier between a metal and semiconductor, which leads to a low output yield in practice^29^. In addition, the lack of vertical momentum and interfacial reflection also block the exportation of SPP-generated carriers^29^. Photons absorbed by SPPs are hence mostly exhausted as heat rather than transformed into exploitable electrical energy.

Here, we consider that the distinct properties of LSPs and SPPs may synergize to produce plasmonic hot carriers. In the weak coupling regime, the interaction between two oscillators only introduces a perturbation to their original properties; thus, SPPs exert little influence on the intrinsic radiative damping of LSPs. However, the energy levels of hybrid polaritons can be greatly altered when oscillators strongly interact with each other in a phenomenon called strong coupling, in which Rabi splitting can be experimentally observed as a distinguishable characteristic of the energy spectrum^30–36^. When the coupling strength exceeds the decoherence rate of the original oscillator, strong coupling occurs^30^, and the energy exchange between the oscillators becomes the dominant relaxation channel. It has been proven that the resonant radiative rate of harmonic oscillators can be modulated in the strong coupling regime^37–39^, which is conducive to addressing the radiation damping bottleneck in the exploitation of hot carriers decayed from LSPs^37^.

In this article, we propose a metal−insulator−metal (MIM) sandwiched heterostructure, where an MoS_2_ monolayer is employed to constitute a Schottky heterojunction with an Au grating on top and serves as an acceptor to harvest hot electrons that decay from LSPs. Stemming from the strong coupling between LSPs and SPPs, hot electron transfer at this heterojunction can be facilitated by coherent energy exchange and the perpendicular enhanced electric field, which decreases the radiative rate of the LSPs and accelerates the exportation of hot electrons. The physical insight presented in this work paves the way to construct plasmonic hot carrier devices with improved performance in the future.

## Results

**Fig. 1:**
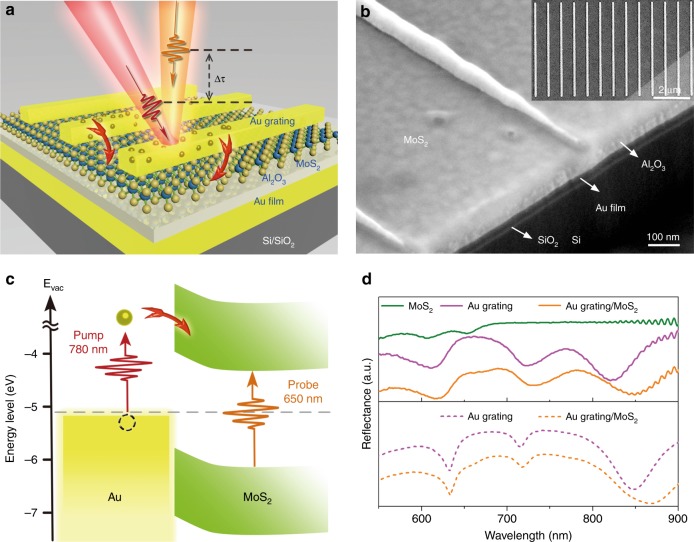
Characterizations of the heterostructure, including an SEM image, a band alignment diagram and reflectance spectra. **a** Schematic of the Au grating/MoS_2_/Al_2_O_3_/Au/Si sandwiched heterostructure. **b** Cross-sectional SEM view of a typical MIM heterostructure, imaged at a 52° tilted angle. The inset shows a top-view SEM image for gratings with a 700 ± 5 nm period. The gray area corresponds to the substrate, while the dark gray represents the MoS_2_ monolayer. **c** The band alignment diagram of the Au grating and MoS_2_ monolayer. Plasmonic hot electrons were pumped at 780 nm, and the transient absorption of the ***A*** exciton in the MoS_2_ monolayer was monitored by a 650 nm probe pulse. **d** Experimentally measured reflectance spectra of the MoS_2_ monolayer, Au grating and Au grating/MoS_2_ at the substrate with a 600 ± 5 nm grating period (solid lines). Reflectance spectra with plasmonic structures were also simulated by the finite-difference time-domain (FDTD) method (dashed lines), which show good agreement with the measurements. SEM scanning electron microscopy

Figure 1a shows a schematic of our Au grating/MoS_2_/substrate sandwiched structure, where the substrate consists of a 20 nm Al_2_O_3_ spacer and a 50 nm Au layer evaporated on a Si/SiO_2_ wafer. Scanning electron microscopy (SEM) images of the heterostructures with different grating periods are shown in Fig. 1b and Figure S1. The electronic band alignment diagram of the Au grating and MoS_2_ monolayer is illustrated in Fig. 1c, which also sketches the transfer process of the plasmonic hot electrons. These electrons are excited by a 780 nm pump laser. After crossing the Schottky barrier, they are injected into the MoS_2_ monolayer underneath and induce a variation in the filled states therein, which is monitored by a 650 nm probe pulse. In the experiment, the 20 nm Al_2_O_3_ layer can prevent hot carriers that decay from SPPs from tunneling into the MoS_2_ monolayer, although most carriers are distributed in the low-energy region and can hardly cross the Schottky barrier. As the energy of the pump laser (1.59 eV) is lower than the bandgap of the MoS_2_ monolayer, excitons cannot be directly excited. Therefore, it is safe to consider that the detected transient absorption signal is only induced by the injection of hot electrons that decay from LSPs, indicating the direct observation of plasmonic hot electron transfer.

Figure 1d shows plots of the measured reflectance spectra of a pristine MoS_2_ monolayer, bare Au grating array and hybrid Au grating/MoS_2_ structure at the substrate. Two absorption peaks were observed at 605 nm (***B*** exciton) and 654 nm (***A*** exciton) for the pristine MoS_2_ monolayer (olive line). Regarding the bare Au grating (magenta line), three obvious resonances appeared at 615, 725, and 823 nm. For the hybrid Au grating/MoS_2_ structure (orange line), the third resonance peak was redshifted by approximately 25 nm due to the change in the surrounding dielectric medium. From the above results, we can see that the third resonance is primarily due to LSPs, while the first two resonances mainly result from SPPs^40^. In fact, all of these resonances are coupled polaritons that hybridize between LSPs and SPPs along either the –*x* (SPP_1_) or +*x* (SPP_2_) axes (Figure S2). In Fig. 1d, the dashed lines are FDTD simulation results for the bare Au grating array and Au/MoS_2_ hybrid structure, which are in good agreement with the experimental results (see Materials and methods for details).

**Fig. 2:**
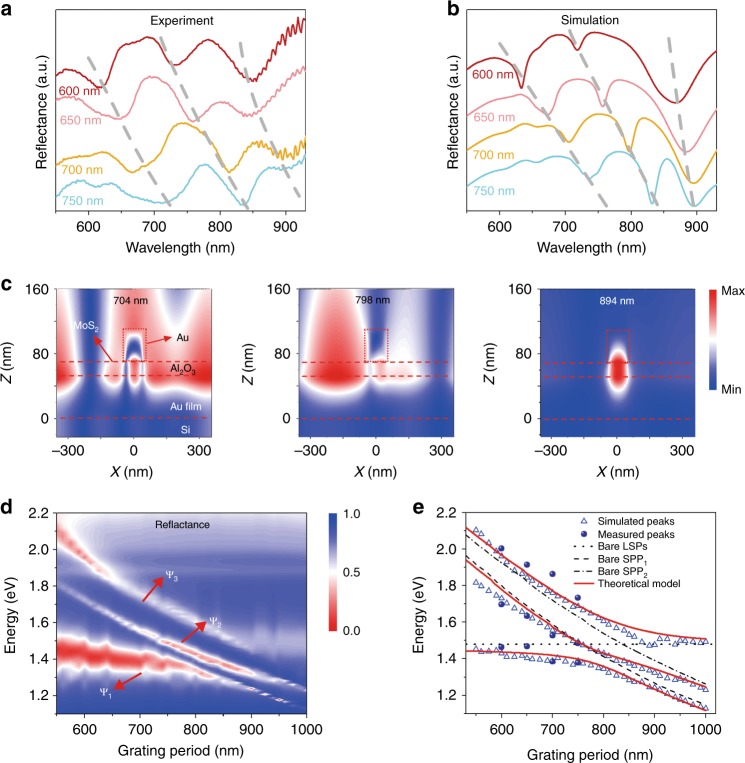
Steady-state reflectance spectra of the sandwiched heterostructures. **a** Measured reflectance spectra of the Au grating/MoS_2_/substrate with grating periods of 600, 650, 700, and 750 nm. **b** Simulated reflectance spectra of the corresponding samples in (**a**). **c** Simulated magnetic field distribution at the resonances for the sample with a 700 nm grating period. **d** The simulated grating-period-dependent reflectance of the sandwiched structures, which exhibits classic Rabi splitting. Ψ_*i*_ (*i* = 1, 2, 3) are eigenstates of the Hamiltonian. **e** Simulated (hollow triangles), measured (filled dots), and theoretical (solid lines) resonance energies as a function of the grating period. The dotted line shows the constant dispersion relation of the uncoupled localized surface plasmons, while the dashed and dash-dotted lines denote the dispersions of the uncoupled SPPs propagating along the –*x* (SPP_1_) and +*x* (SPP_2_) axes, respectively. SPP surface plasmon polariton

It is well known that a steady-state reflectivity measurement is an efficient way to investigate strong coupling. Figure 2a, b shows the measured and simulated reflectance spectra of Au/MoS_2_ hybrids with Au grating periods ranging from 600 to 750 nm, where the three plasmon resonances behave in a similar fashion. The magnetic field distribution of these resonances for a grating period of 700 nm was calculated in Fig. 2c. It is apparent that the mode at 704 nm (798 nm) is dominated by SPPs propagating along the +*x* (−*x*) axis, and the third resonance is mainly due to LSPs. Similar results can be obtained from the electric field distribution in Figure S3 and Supplementary Section 1.

Figure 2d shows the simulated reflectance mapping for grating periods ranging from 550 to 1000 nm, where two clear energy anticrossings appear. These avoided crossings correspond to Rabi splitting and demonstrate strong coupling between the uncoupled modes. A coupled oscillator model was successfully used to study the observed strong coupling^41–43^. In our case, the Hamiltonian *H* can be written as

1 $$\left( {\begin{array}{*{20}{c}} {E_{{\mathrm {LSPs}}}} & {V_1} & {V_2} \\ {V_1^ \ast } & {E_{{\mathrm {SPP}}_{ 1}}} & {V_3} \\ {V_2^ \ast } & {V_3^ \ast } & {E_{{\mathrm {SPP}}_{ 2}}} \end{array}} \right)$$

where *E*_LSPs_, *E*_SPP1_ and *E*_SPP2_ are the resonance energies of the uncoupled LSP, SPP_1_, and SPP_2_ states, and *V*_*i*_ (*i* = 1, 2, 3) represent the coupling strengths between these states. Ψ_*i*_ (*i* = 1, 2, 3) are defined as eigenstates of *H* with the lowest, middle, and highest eigenenergies. They are also mixtures of uncoupled modes and can be expressed as

2 $$\Psi _i = c_{i1}\left| {{\mathrm {LSPs}}} \right\rangle + c_{i2}\left| {{\mathrm {SPP}}_{ 1}} \right\rangle + c_{i3}\left| {{\mathrm {SPP}}_{ 2}} \right\rangle$$

where *c*_*ij*_ (*j* = 1, 2, 3) are coefficients.

To solve the Hamiltonian, the dispersion relations of the uncoupled LSPs, SPP_1_, and SPP_2_ are plotted in Fig. 2e as dotted, dashed, and dash-dotted lines, respectively, from which we can see that the LSP resonance energy (*E*_LSPs_ = 1.46 eV) remains constant as the grating period changes. For the uncoupled SPP modes, their dispersion relations can be obtained from the equation

3 $$\pm K_{{\mathrm {spp}}} = K_x \pm m\frac{{2\pi }}{P}$$

where $K_{{\mathrm {spp}}} = \frac{\omega }{c}\sqrt {\frac{{\varepsilon _1\varepsilon _2}}{{\varepsilon _1 + \varepsilon _2}}}$ is the SPP wavevector, *ε*_1_ (*ε*_2_) is the dielectric permittivity of the Au (dielectric) layer, $K_x = \frac{\omega }{c}\sin \theta$ is the horizontal wavevector component of the incident light, *m* is an integer and *P* corresponds to the grating period. For such an air/Al_2_O_3_/Au interface, the dispersion relation of the SPPs cannot be directly described by a classic model of two semi-infinite layers. However, the actual dispersion can be acquired from a modified model, in which an effective medium with a wavelength-dependent refractive index is introduced (Figure S4 and Supplementary Section 2).

With the fitting parameters *V*_*i*_ (*i* = 1, 2, 3) equal to 0.075, 0.075, and 0.06 eV, respectively, theoretical results based on the coupled oscillator model were calculated. The results are shown as red solid lines in Fig. 2e. *V*_1_ = *V*_2_ means that the coupling strength between the LSPs and SPP_1_ equals that between the LSPs and SPP_2_, which may be attributed to *m* = 1 for both SPP_1_ and SPP_2_. The simulated (hollow triangles), measured (filled dots), and theoretical (solid lines) results are in excellent agreement, as demonstrated in Fig. 2e, which implies that strong coupling between the LSPs and SPPs occurs in our proposed MIM heterostructures.

**Fig. 3:**
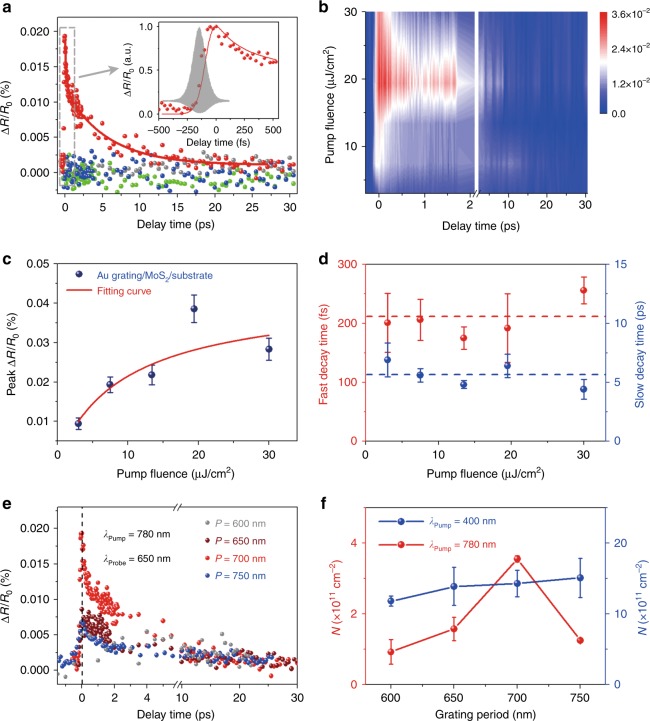
Transient absorption spectra of the heterostructures for different grating periods and pump wavelengths. **a** Differential reflection spectra of the Au grating/MoS_2_/substrate (red dots) and control samples of the Au grating, MoS_2_ monolayer and substrate for a pump fluence of 7.5 μJ/cm^2^. The red line is a fitting curve for the descent of Δ*R*/*R*_0_(*t*) with a biexponential function. The inset shows details of the rising process with normalization. The data shown in gray represent the interferometric autocorrelation function for the pump pulses, from which the upper envelope with a full width at half-maximum of ~130 fs is taken as the IRF. The red line in the inset was obtained by convoluting the IRF and the actual electron injection function. **b** Pump-fluence-dependent transient absorption spectra of the heterostructure. The scale bar corresponds to the intensity of the ultrafast signals. **c** The maximum amplitude of Δ*R*/*R*_0_(*t*) as a function of pump fluence. The red line is a fitting curve with the saturation formula of Eq. (4). **d** Fast and slow decay lifetimes varying with pump fluence. **e** Ultrafast pump-probe spectra of the Au grating/MoS_2_/substrate for different grating periods pumped at 780 nm with 7.5 μJ/cm^2^. **f** Excited (injected) electron densities derived from the peak amplitudes of Δ*R*/*R*_0_(*t*). The red dots represent the grating-period-dependent densities of electrons injected into the MoS_2_ monolayer pumped at 780 nm, while the blue dots correspond to the densities of electrons excited directly at the pump wavelength of 400 nm. IRF instrument response function

To investigate the dynamics of plasmonic hot electron transfer in the strong coupling regime between the LSPs and SPPs, femtosecond pump-probe measurements in a reflection configuration were carried out. Figure 3a presents differential reflection spectra Δ*R*/*R*_0_(t) of the heterostructure with a 700 nm Au grating period (red dots) and reference samples, which include a bare Au grating array on the substrate (blue), a pristine MoS_2_ monolayer on the substrate (green) and the substrate (gray). The pump had a wavelength of 780 nm and a fluence of 7.5 μJ/cm^2^, and 650 nm probe pulses with a fluence of 0.75 μJ/cm^2^ were used to detect the transient absorption of the ***A*** exciton in the MoS_2_ monolayer. For the bare Au grating array on the substrate, no transient differential reflection signal appears, proving that plasmonic hot electron relaxation cannot induce ultrafast signals in this case. One of the causes of this phenomenon could be the low pump and probe fluences in our experiments, which are two orders of magnitude weaker than those used in typical pump-probe measurements of metallic electron relaxations^44^. This reason is verified in Figure S5 and Supplementary Section 3, in which the pump fluence is enhanced by two orders of magnitude. The other reason is the pump wavelength of 780 nm pump, which is chosen to excite SP resonances rather than interband transitions of Au that have larger absorption cross-sections^44^.

The ultrafast signal only emerges in the Au grating/MoS_2_/substrate heterostructure, revealing that the variation in the occupancy in MoS_2_ arises from plasmonic hot electron injection. As the injected hot electrons fill unoccupied states in the conduction band of MoS_2_ and rapidly relax to the exciton level, the absorbance of the ***A*** exciton with the pump is lower than that without the pump. Thus, the transfer process can be represented by the rising edge of Δ*R*/*R*_0_(*t*), as shown in the inset. The injection time was estimated to be approximately 40 fs by deconvoluting the signal with the instrument response function (IRF) (see Supplementary Section 5 for the detailed deconvolution process). The red line in the inset was obtained by convoluting the IRF and the actual electron injection function, which can well reproduce the experimentally measured transient differential signal. Then, the decay of transferred hot electrons occurs during the descent of Δ*R*/*R*_0_(*t*). The decay process can be well fitted with a biexponential function and is caused by electron−electron and electron−phonon scattering.

The pump fluence dependence of the ultrafast signal was studied in Fig. 3b, and the maximum amplitudes of the spectra were extracted in Fig. 3c. The peak amplitude increases and gradually saturates as the fluence increases, and the relationship can be fitted by

4 $$\Delta R/R_0(0) \propto \frac{f}{{f + f_{{\mathrm {sat}}}}}$$

where *f* and *f*_sat_ are the pump fluence and the saturation value, respectively. When the fluence exceeds 10 μJ/cm^2^, the peak of Δ*R*/*R*_0_(*t*) is greatly influenced by the saturation effect. This phenomenon could be caused by a mass accumulation of transferred hot electrons because these electrons repel like charges and prevent further injection into the MoS_2_ monolayer. Different from the saturating trend of the peak amplitude, the decay processes in the transient absorption spectra are independent of the pump fluence, as shown in Fig. 3d, and can be fitted by biexponential functions with average parameters of 210 fs and 5.5 ps, which are attributed to the lifetimes of electron−electron and electron−phonon interactions.

Apart from the transfer timescale, the injected hot electron density is another important factor. As ultrafast signals arise from variations in occupation number in the MoS_2_ monolayer, the largest intensity of Δ*R*/*R*_0_(*t*) can in principle represent the density of excited (injected) electrons. At a pump wavelength of 780 nm, ultrafast signals arise from the injection of plasmonic hot electrons. As a result, the densities of the electrons transferred into MoS_2_ cannot be directly evaluated by the absorbance of MoS_2_. To derive the densities in this case, a quantitative relationship between the densities and peak amplitudes of Δ*R*/*R*_0_(t) needs to be established first. To obtain this relation, transient absorption spectra pumped at 400 nm were measured, because ultrafast signals originate from the direct excitation of electrons in the valence band of MoS_2_ in this case. Therefore, the excited electron density can be calculated from the absorbance of the MoS_2_ monolayer. At a pump wavelength of 400 nm with 7.5 μJ/cm^2^, the excited electron density equals 1.47 × 10^12^ cm^−2^ with a corresponding peak amplitude of Δ*R*/*R*_0_(*t*) of 0.08% (Figure S6). Based on this result, a linear relationship between the density and peak amplitude was eventually established (see Supplementary Section 6 for details). The density of injected hot electrons pumped at 780 nm, which was derived from the peak amplitude in Fig. 3a, was estimated as 3.55 × 10^11^ cm^−2^.

Moreover, to further confirm the active role of SPs in injecting hot electrons, ultrafast transient absorption measurements of samples pumped at 780 nm for various grating periods were performed, as shown in Fig. 3e. Based on their peak amplitudes, the transferred electron densities were calculated similarly in Fig. 3f. The density reaches a maximum for a grating period of 700 nm because strong coupling occurs therein and the resonance peak of Ψ_2_ (the eigenstate with the middle eigenenergy) is closer to the pump wavelength than the resonances of the other grating periods (Fig. 2a). In contrast, the excited electron density hardly changes with the grating period when the pump wavelength is 400 nm, as illustrated in Fig. 3f and Figure S7, in which case the plasmonic modes cannot be resonantly excited. This distinct grating period dependence at different pump wavelengths is ascribed to the diverse origins of the occupancy number variation in MoS_2_.

From the above results, plasmonic hot electron transfer from the Au grating to the MoS_2_ monolayer was directly observed in the strong coupling regime, in which the injection time and density were approximately 40 fs and 3.55 × 10^11^ cm^−2^, respectively, for a pump fluence of 7.5 μJ/cm^2^.

**Fig. 4:**
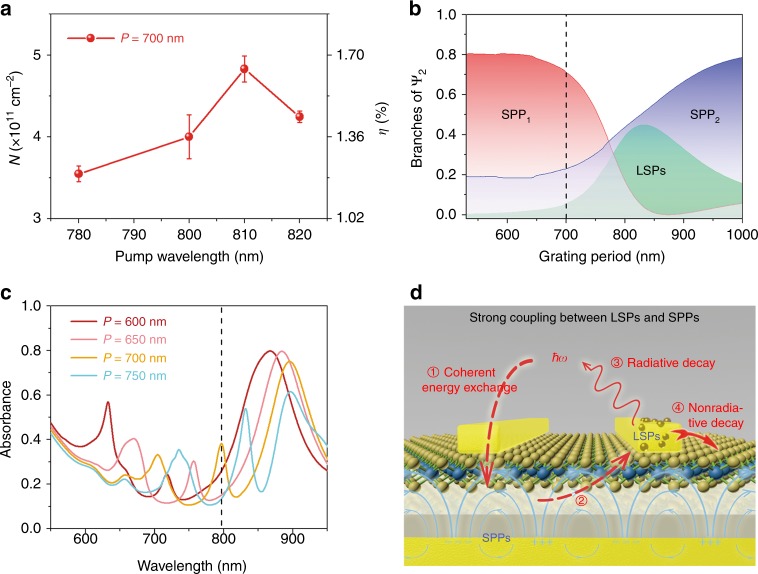
Evidence for confirming the effect of strong coupling and the proposed model. **a** Transferred hot electron densities and corresponding *η* for the heterostructure with a 700 nm grating period at different pump wavelengths. **b** Branches of Ψ_2_ (|*c*_21_|^2^, |*c*_22_|^2^, and |*c*_23_|^2^) as a function of the grating period. **c** The simulated absorbance of the heterostructures with grating periods of 600, 650, 700, and 750 nm. For a grating period of 700 nm, the absorbance is approximately 0.4 when Ψ_2_ is resonantly excited. **d** A diagrammatic model proposed to describe the physical processes in the strong coupling regime between LSPs and surface plasmon polaritons. Coherent energy exchange is represented by the dashed lines labeled by ① and ②. The processes labeled as ③ and ④ denote the radiative and nonradiative relaxation channels of the LSPs, respectively. LSP localized surface plasmon

The injection efficiency is an important factor in carrier transfer studies. In our work, there is also significant evidence to prove the contribution of strong coupling in advancing plasmonic hot electron transfer. Here, we reveal the effect of strong coupling by comparing experimental and theoretical electron injection yields. The external quantum yield *η* is defined as the ratio of transferred electron densities to incident photon densities. To obtain a maximum yield in the experiment, the pump wavelength was altered, as shown in Fig. 4a. The resonance wavelength of Ψ_2_ is 813 nm for the 700 nm grating period, and the peak of *η* follows the resonance, reaching its largest value of 1.65% at the pump wavelength of 810 nm.

To estimate theoretical efficiencies, we first calculated all branches of Ψ_*i*_ (*i* = 1, 2, 3) (|*c*_*ij*_|^2^) for different grating periods in Fig. 4b and Figure S8 according to the coupled oscillator model. For a grating period of 700 nm, the LSP branches (|*c*_*i*1_|^2^) are approximately 85, 6, and 10% in Ψ_*i*_ (*i* = 1, 2, 3), respectively, in agreement with the magnetic field distribution in Fig. 2c. Since Ψ_2_ is near-resonant with a 780 nm pump, its fractions are analyzed in detail in Fig. 4b. It is interesting to note that SPP_1_ dominates Ψ_2_ and the LSPs only contribute 6% for a 700 nm grating period, which implies that the LSPs are launched with 6% of the energy in Ψ_2_.

Next, we evaluate the theoretical injection efficiency of hot electrons induced by the LSPs individually. Before transferring into MoS_2_, many steps must occur for the LSPs to generate hot electrons. First, pump pulses couple to Ψ_2_ with an absorbance of approximately 40% (Fig. 4c), and only 6% of the coupled energy is used to stimulate LSPs. In practice, both hot electrons and holes are excited, all of which are distributed in a range of energy and momentum. Assuming that hot electrons attain half of the energies and all are injected into MoS_2_, the efficiency is 1.2% (=40% × 6% × 50%) when considering the steps up to now, which equals the measured yield when pumped at 780 nm. However, in fact, only energetic hot electrons with enough vertical momentum are capable of crossing the Schottky barrier^17,29^. In addition, they must overcome interfacial reflection and recombination with defects at the interface before finally transferring into MoS_2_. This comparison result suggests that *η* should much less than the measured value if hot electrons are solely produced by the LSPs.

Based on the analysis above, we propose a model to depict the mechanism as illustrated in Fig. 4d, in which strong coupling between plasmonic modes is the ultimate reason for the elevated *η*. In the strong coupling regime, the energies of uncoupled oscillators are coherently exchanging^31^. In this case, photons emitted by the radiative damping of LSPs are reabsorbed into SPPs by coherent energy exchange (①). As uncoupled SPPs with prolonged lifetimes are almost relaxed nonradiatively, their captured energies can be stored for a relatively long time. Thereafter, the intrinsic energies of the SPPs and reabsorbed energies can also be delivered to the LSPs through coherent energy exchange (②), experiencing radiative (③) and nonradiative (④) relaxations once again. The net result is that the original energies stored in the SPPs and radiation energies of the LSPs are recycled to produce hot carriers rather than being exhausted as heat or radiating into free space directly, such that the inaccessible energies in the SPPs are utilized and radiative damping in the LSPs is suppressed. In the strong coupling regime, the enhanced electric field surrounding MoS_2_ at resonance is another element that facilitates hot electron transfer. The electric field induced by image charges in the Au grating and Au film provides hot electrons with requisite vertical momenta for crossing the Schottky barrier. In such a picture, LSPs and SPPs collaborate excellently to take full advantage of unavailable energies for generating hot carriers in the strong coupling regime.

## Discussion

In summary, by virtue of femtosecond pump-probe spectroscopy, direct plasmonic hot electron transfer from an Au grating to an MoS_2_ monolayer was successfully observed in an MIM structure in the strong coupling regime. Plasmonic hot electron transfer occurs at ~40 fs with a maximum *η* of 1.65%. Strong coupling between LSPs and SPPs generates plasmonic hot electrons from energy and momentum. Coherent energy exchange allows photons radiated by the decay of LSPs to be reabsorbed by SPPs and originally unavailable energies stored in SPPs to participate in generating hot carriers. In this picture, low-loss SPPs that are nonradiative in nature function as an “energy recycle bin”: capturing, storing and delivering radiative energies of the LSPs. Due to the intense electric field induced by image charges in the MIM structure, the perpendicular momenta required for crossing the Schottky barrier are provided. Ascribed to strong coupling, the complementary aspects of LSPs and SPPs overcome the intrinsic drawbacks of individual plasmonic modes in exploiting hot carriers. The insight presented in this work is also applicable to other metal-2D semiconductor, metal-molecular and metal-organic semiconductor systems. The spectral range of strong coupling can also be modulated in the near-infrared region by tuning the geometry and size, which holds great prospects for improving the performance of plasmonic hot carrier devices in fields involving photoconversion.

## Materials and methods

### Sample preparations

The Au film (50 nm) and Al_2_O_3_ layer (20 nm) were deposited on an Si/SiO_2_ wafer by electron beam evaporation sequentially. The CVD-grown MoS_2_ monolayer was then transferred onto the prepared substrate with the wetting transfer method. The MoS_2_ monolayer grown on an Si/SiO_2_ wafer was first spin-coated with poly(methyl methacrylate) (PMMA) and baked on a hot plate (180 °C) for 5 min. It was then etched in KOH solution for 6 h to strip the film from the wafer. Next, the film was removed and transferred to deionized water to rinse off impurities. This cleaning process was repeated three times. The film was then transferred onto the prepared substrate and heated for 5 min on a 100 °C hot plate. The PMMA on the film was dissolved with acetone steam that was produced by heating acetone at 150 °C. An MoS_2_ monolayer was eventually left on the substrate. Au gratings 40 nm in thickness and 85 ± 5 nm in width were fabricated using electron beam lithography and electron beam evaporation methods. To obtain a cross-sectional SEM image, the heterostructure was etched by a focused ion beam.

### Reflectance measurements

A hyperspectral imaging system (Cytoviva HISV3) was adapted to perform the reflectance measurements. Reflectance spectra were recorded by the spectrometer (Horiba iHR550) with a ×10 objective (Olympus MPlanFL, NA = 0.25). Relative reflectivity was utilized to represent the reflectance spectra, which was obtained by dividing the reflected intensity of the sample by that of the substrate. Nonpolarized white light passed through a linear polarizer (Thorlabs LPNIRE100-B) with the polarization direction along the *x*-axis. As the normal incident white light was confined by the objective, the in-plane wavevector *k*_*x*_ was not zero despite the value being small compared with the momentum provided by gratings. However, this issue could not be neglected as it resulted in a splitting of uncoupled SPP modes (SPP_1_ and SPP_2_).

### Finite-difference time-domain (FDTD) simulations

The FDTD method was employed to simulate the reflectance spectra and electromagnetic field distributions of heterostructures. The relative permittivities of the Au and MoS_2_ monolayers were taken from the literature^45,46^, and the refractive index of Al_2_O_3_ was taken as 1.77. The incident angle of TM-polarized light was set at 3° because oblique incidence with an angle of *θ* ~ 3° can supply a similar in-plane wavevector to simulate the influence of objective confinement. Bloch boundary conditions were used in the *x* direction, symmetric boundaries were applied in the *y* direction and perfectly matched layer boundaries were used in the *z* direction.

### Femtosecond pump-probe measurements

Typical femtosecond transient absorption spectra measurements in a reflection configuration were carried out. The mode-locked oscillator (Tsunami 3941C-25XP) generated 800 nm femtosecond pulses with a repetition rate of 80 MHz and a pulse duration of ~73 fs. The output laser was split into two parts. One part was used as the pump and chopped at 1500 Hz, while the other part was focused onto photonic crystal fiber (Newport SCG-800) to generate supercontinuum white light extending from 550 to 1400 nm. The probe was then selected with a 650 ± 10 nm bandpass filter (Thorlabs). The pump pulses passed through a linear polarizer (Thorlabs LPNIRE100-B), with the polarization direction along the *x* axis. The reflected probe pulses were collected by a high-sensitivity photomultiplier (Thorlabs PMM02) and were converted into electric signals. The spot size of the focused probe was approximately 2 µm. The delay time between the pump and probe pulses was controlled by a stepper linear stage (Newport M-ILS150PP). Differential reflection signals ∆*R/R*_0_(*t*) *=* (*R* *−* *R*_0_)/*R*_0_ were acquired by subtracting the reflectivity of the probe with pump pulses (*R*) from that without the pump (*R*_0_).

## Supplementary Information

Supporting information
