# Supplementary material for: Direct observation of ultrafast plasmonic hot electron transfer in the strong coupling regime
Source: Light Sci Appl. 2019 Jan 16;8:9. doi: 10.1038/s41377-019-0121-6 (PMC6333624; doi:10.1038/s41377-019-0121-6)
Supplement: Supplementary file 1 — Supporting information [file 41377_2019_121_MOESM1_ESM.docx]

Supporting information for

**Direct Observation of Ultrafast Plasmonic Hot Electron Transfer in the Strong Coupling Regime**

Hangyong Shan^1,*^, Ying Yu^1,*^, Xingli Wang^2^, Yang Luo^1^, Shuai Zu^1^, Bowen Du^1^, Tianyang Han^1^, Bowen Li^1^, Yu Li^1^, Jiarui Wu^1^, Feng Lin^1^, Kebin Shi^1^, Beng Kang Tay^2,3^, Zheng Liu^2,3^, Xing Zhu^1^, Zheyu Fang^1,†^

^1^School of Physics, State Key Lab for Mesoscopic Physics; Academy for Advanced Interdisciplinary Studies; Collaborative Innovation Center of Quantum Matter, Peking University, Beijing 100871, China.

^2^CNRS International-NTU-Thales Research Alliance (CINTRA), Nanyang Technological University, Singapore 637553, Singapore.

^3^Centre for Micro-/Nano-electronics (NOVITAS), School of Electrical and Electronic Engineering; Centre for Programmed Materials, School of Materials Science and Engineering, Nanyang Technological University, Singapore 637553, Singapore.

^*^These authors contributed equally to this work.

^†^Correspondence author. E-mail: [zhyfang@pku.edu.cn](mailto:zhyfang@pku.edu.cn)


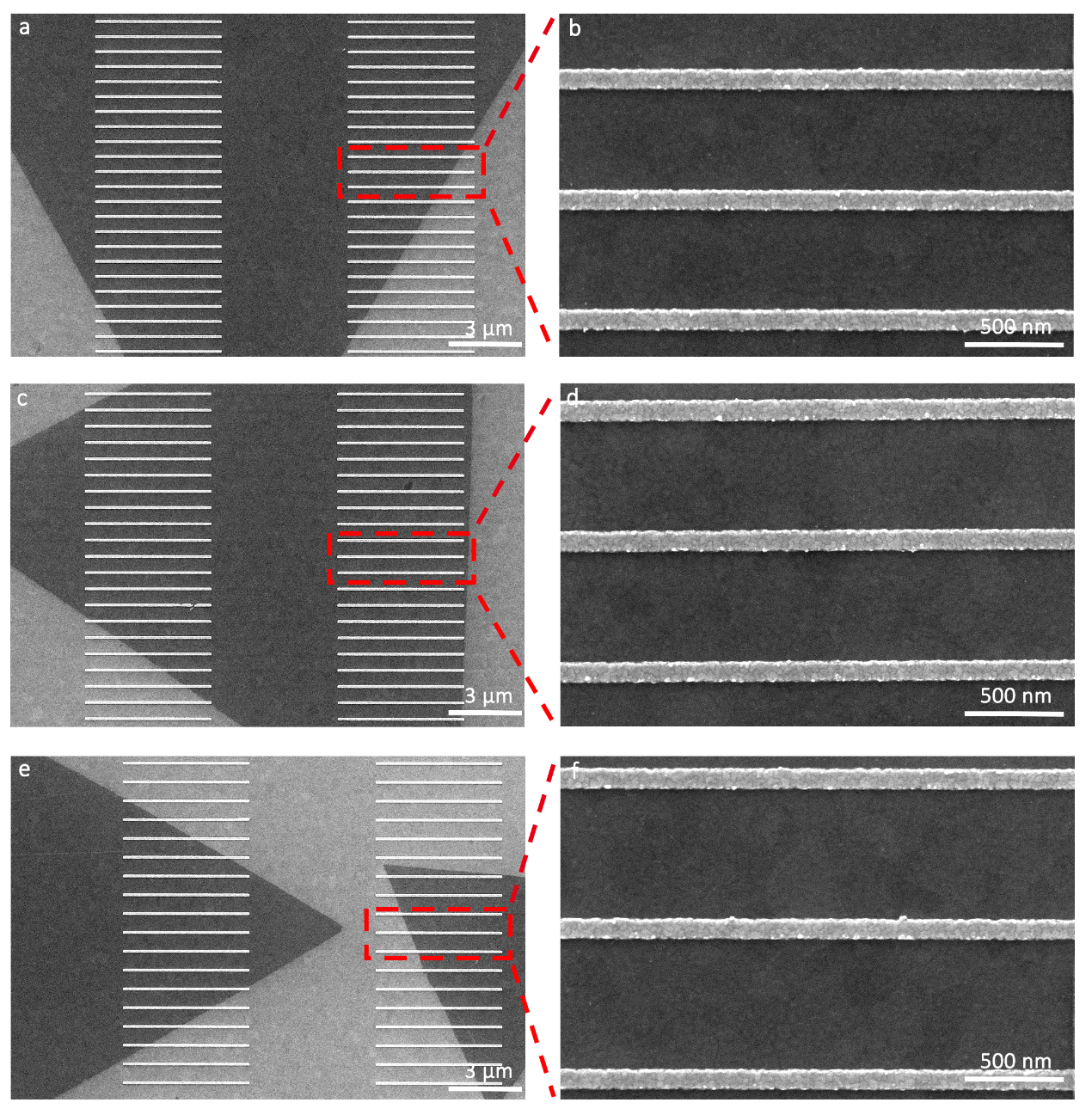


**Figure S1.** SEM images of MIM sandwiched heterostructures. (a), (c) and (e) show top-view SEM images for sandwiched structures with Au grating periods of 600, 650 and 750 nm, where the scale bar is 3 µm. (b), (d) and (f) demonstrate corresponding amplified images with the scale bar of 500 nm.


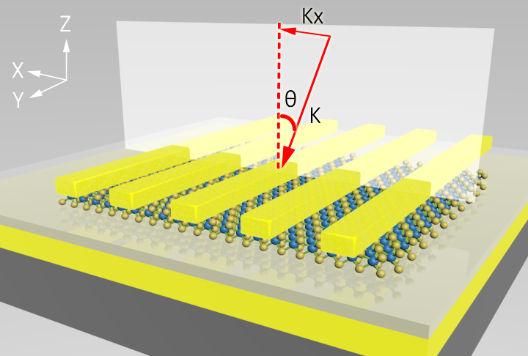


**Figure S2.** The schematic view of FDTD simulation. In FDTD simulations, the TM-polarized light with incident angle *θ* ~ 3° is linearly polarized, of which the main electric field component is perpendicular to the Au grating array (along the *x* axis).

**Section 1. The electric field distribution at resonant wavelengths.**

Figure S3 displays electric field distribution at three resonant peaks for the period of 700 nm. Propagating character is apparent at the first two modes (704 and 798 nm). This indicates that the first two resonances are primarily contributed by SPPs, while the third one is dominated by LSPs. For the mode at 798 nm, the electric field surrounding MoS_2_ monolayer is enhanced by an order of magnitude, which facilitates the hot electron transfer from momentum.


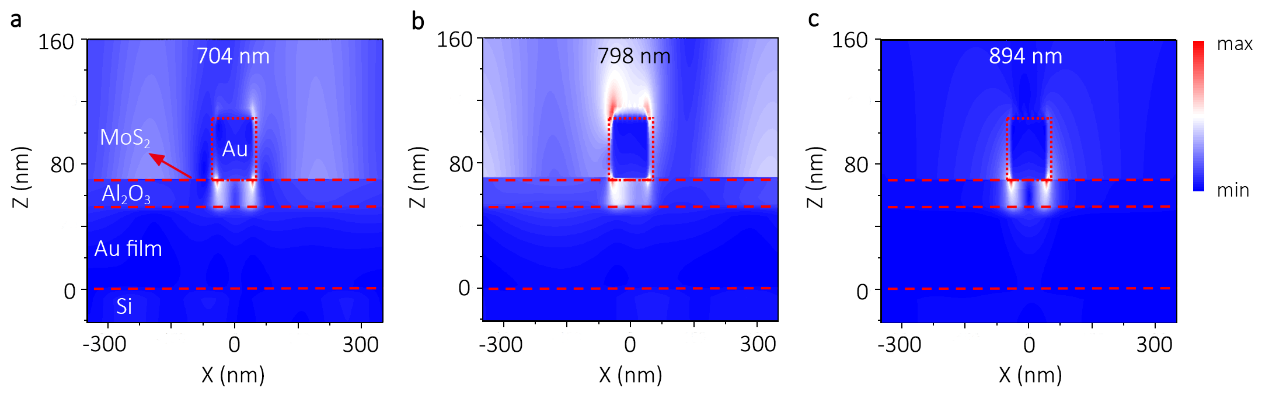


**Figure S3.** The simulated electric field distribution at resonances for the sample with 700 nm period. It can be seen that SPPs modes are obvious at resonances of 704 and 798 nm, while LSPs dominate the third mode (894 nm).

**Section 2. The refractive index of effective medium and dispersion relations of uncoupled SPPs.**

To solve the Hamiltonian, dispersion relations of uncoupled SPPs should be settled. In our structures, there is a 20 nm Al_2_O_3_ spacer between the Au film and air (air/Al_2_O_3_/Au interface), which can affect the dispersion of SPPs. Although dispersion relations of SPPs at the air/Al_2_O_3_/Au interface cannot be directly described by the ideal two-layer model (air/Au interface), the actual wave vector can be obtained from a modified one. In the revised model, the air/Al_2_O_3_ layer is substituted by a semi-infinite dielectric layer (effective medium), which forms effective medium/Au interface as shown in Figure S4a. The wave vector of SPPs at the air/Al_2_O_3_/Au interface equals to that at the effective medium/Au interface. As long as the refractive index of effective medium is acquired, dispersion relations of SPPs at the effective medium/Au interface can be analytically solved by the two-layer model. Thereafter, wave vector of SPPs at the air/Al_2_O_3_/Au interface can be obtained.

Based on FDTD simulations, the refractive index of effective medium at different wavelengths are calculated and shown in Figure S4b. The value of effective refractive index is analogous to that of air, implying that the 20 nm Al_2_O_3_ film makes a small influence on the dispersion relation when inserting into the air/Au interface.

With the effective refractive index, the relationship between the grating period and the energy of SPPs is derived, shown as dashed and dash-dotted lines in Figure 2e. To obtain the full characterization of dispersion relations for SPPs at the air/Al_2_O_3_/Au interface, the relation between the grating period and the momentum of SPPs should be known, which can be derived from Equation (3). With the equation, we indenpendently calculate dispersion relations with the data of bare SPP_1_ and SPP_2_ modes illustrated in Figure 2e. Two calculated dispersion relations are shown in Figure S4c, which are perfectly consistent. Certainly, the dispersion of SPPs is merely determined by ε_1_ and ε_2_ (the dielectric permittivity of Au and effective medium), and is indenpendent of the propagation direction.

**
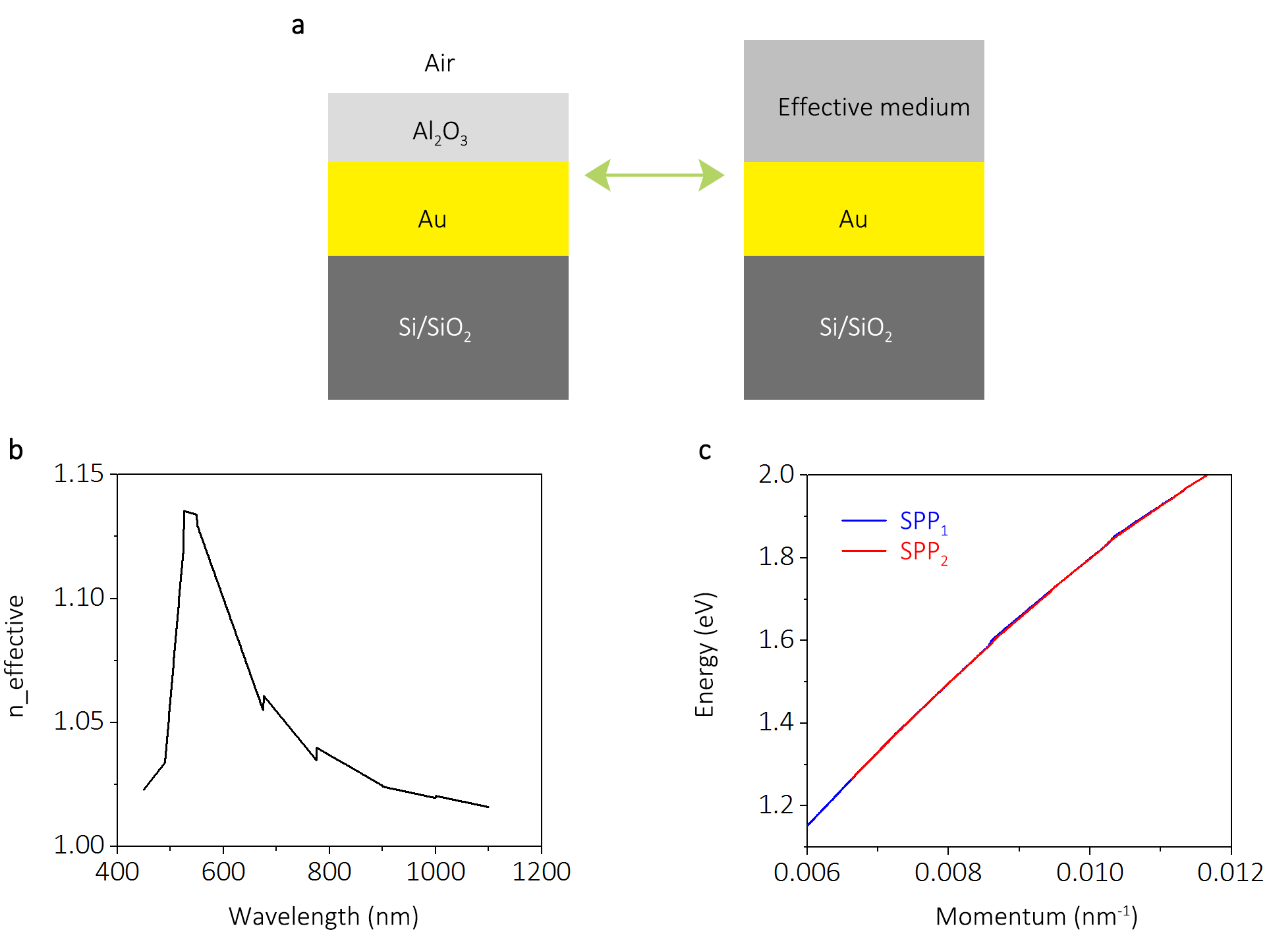
**

**Figure S4.** The method to analytically solve dispersion relations of SPPs at the air/Al_2_O_3_/Au interface. (a) The schematic of the effective medium. (b) The refractive index of the effective medium calculated by FDTD methods. The discontinuity is caused by the piecewise fitting over the wavelength range, of which the impact on dispersion relations can be nearly ignored. (c) Dispersion relations of SPPs at the air/Al_2_O_3_/Au interface, derived from bare SPP_1_ and SPP_2_ modes separately.

**Section 3. The transient signal of the bare Au grating array at high pump fluence.**

For the bare Au grating array on the substrate, no transient differential reflection signal appears when the pump and probe fluences are 7.5 and 0.75 μJ/cm^2^ respectively. This result should be aroused by low pump and probe fluences.

To further verify this inference, we measured the transient signal of the bare Au grating with 700 nm period on the substrate at high fluences. The pump laser was used to resonantly excite surface plasmons and the wavelength was tuned to 810 nm, while the probe wavelength was 650 nm. The pump and probe fluences are increased to 1200 and 7.5 μJ/cm^2^, respectively. In this case, the free electron excitation and relaxation results in a transient differential reflection signal, as shown in Figure S5.

In this paper, the pump fluence is 7.5 μJ/cm^2^ if not specifically stated. Under this fluence, the free electron excitation and relaxation of bare Au grating cannot induce transient signals. Therefore, there is no need to consider this effect when analyzing transient signals of the Au grating/MoS_2_/substrate heterostructure.

**
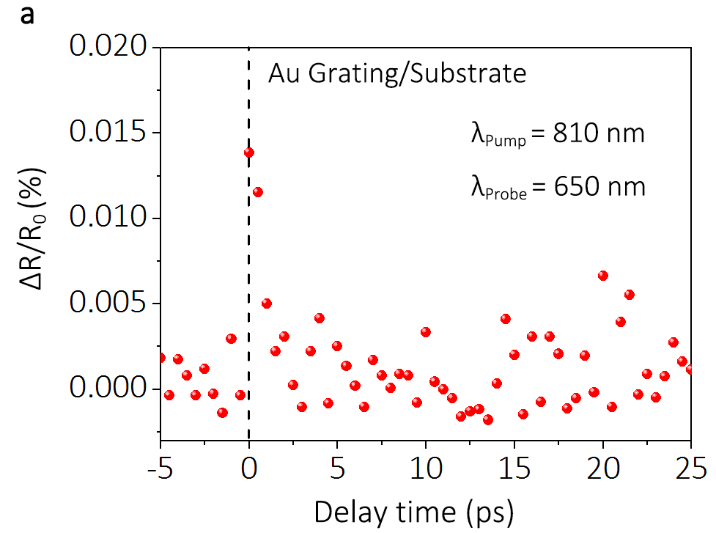
**

**Figure S5.** The transient signal of the bare Au grating on the substrate. The fluences of 810 nm pump and 650 nm probe are 1200 and 7.5 μJ/cm^2^. This transient signal is caused by the free electron excitation and relaxation.

**Section 4. The discussion of the band gap for SPPs.**

When the metal-dielectric interface is modulated with a periodically corrugated surface and the Bragg vector (2π/period) is twice of the wave vector of SPPs (*K_SPP_*), modes propagating along the direction of Bragg vector can be scattered by the surface to form standing waves. Two possible standing waves with different energies can be formed, which have different electromagnetic field and charge distributions. Therefore, an energy gap opens up in the dispersion of SPPs.

However, to experimentally observe such an energy gap, special methods should be employed to realize the excitation of SPPs (momentum matching) and the creation of SPPs band gap. One method is using the prism coupling technique to excite SPPs and the periodical surface (the period equals to half the wavelength of SPPs) to form an energy gap.[^1^](#_ENREF_1),[^2^](#_ENREF_2) The other method is to employ double periodical surfaces, in which one corrugation with wave vector *K* couples photons to SPPs and the second corrugation with wave vector 2*K* provides the Bragg scattering to form the energy gap.[^3^](#_ENREF_3),[^4^](#_ENREF_4)

In our work, the grating is only utilized to meet the momentum matching condition of exciting SPPs. The energy gap of SPPs cannot be observed in experiments nor affects the Rabi splitting.

**Section 5. The deconvolution process for the rising edge of ΔR/R_0_(t).**

The measured rising time of ΔR/R_0_(t) (~250 fs, inset of Figure 3a) is limited by the laser pulse duration that can be represent by the instrument response function (IRF). The measured rising edge is actually obtained by convoluting the IRF and the actual electron injection function. As the actual injection function can be expressed as$\exp(t/\tau)$, where *τ* is the electron injection time, the experimentally measured transient signal equals to $IRF\otimes\exp(t/\tau)$. To reduce the influence of pulse duration and get the actual transfer time *τ*, the deconvolution process should be performed.[^5-7^](#_ENREF_5)

**Section 6. The derivation of transferred hot electron density at the pump wavelength of 780 nm.**

The largest intensity of ultrafast signal for MoS_2_ monolayer can manifest the density of excited (injected) electrons. Hence, in order to estimate electron densities transferred into MoS_2_ at the pump wavelength of 780 nm, a quantitative relationship between the density and peak amplitude of ΔR/R_0_(t) needs to be established. To get this relation, transient absorption measurements pumped at 400 nm were performed (Figure S6a), because excited electron densities can be straightly derived in this case. Detailed derivation processes can be divided into three steps as following:

1. First, we calculated excited exciton (electron) densities pumped at 400 nm. Provided that every absorbed photon excites one exciton in the MoS_2_ monolayer, the 400 nm pump pulse at 7.5 μJ/cm^2^ can excite electrons with a density of 1.47×10^12^ cm^-2^ when the absorption coefficient is 1.5×10^6^ cm^-1^ for MoS_2_ monolayer.[^8^](#_ENREF_8),[^9^](#_ENREF_9) Similarly, densities at different pump fluences were calculated, plotted as the dashed line in Figure S6b.
2. Secondly, we established the quantitative relation between the density and peak amplitude of ΔR/R_0_(t) by the results pumped at 400 nm. Figure S6a shows pump fluence-dependent transient absorption spectra of the heterostructure with 700 nm period, in which the pump wavelength was 400 nm. The peak amplitude of ΔR/R_0_(t) was 0.08% at 7.5 μJ/cm^2^. A linear relationship between the density *N* and peak of ΔR/R_0_(t) was supposed *N* = *k* × ΔR/R_0_(0), where *k* is a constant, and corresponding values (1.47×10^12^ cm^-2^ and 0.08%) under 7.5 μJ/cm^2^ were taken as criteria.[^10^](#_ENREF_10),[^11^](#_ENREF_11) Based on this linear relation, electron densities obtained from ΔR/R_0_(t) are plotted as solid points in Figure S6b. It can be seen that the deviation between directly calculated density and that estimated from measured ΔR/R_0_(0) can be ignored when the pump fluence is lower than 20 μJ/cm^2^ (Figure S6b), which indicates that the assumed linear relation is effective to derive electron densities pumped at 780 nm with 7.5 μJ/cm^2^.
3. Thirdly, transferred electron densities pumped at 780 nm were calculated. Under the fluence of 7.5 μJ/cm^2^, the peak value of ΔR/R_0_(t) was 0.0193% as shown in Figure 3a. The injected electron density was then estimated to be 3.55×10^11^ cm^-2^ according to the linear relationship derived in (2).


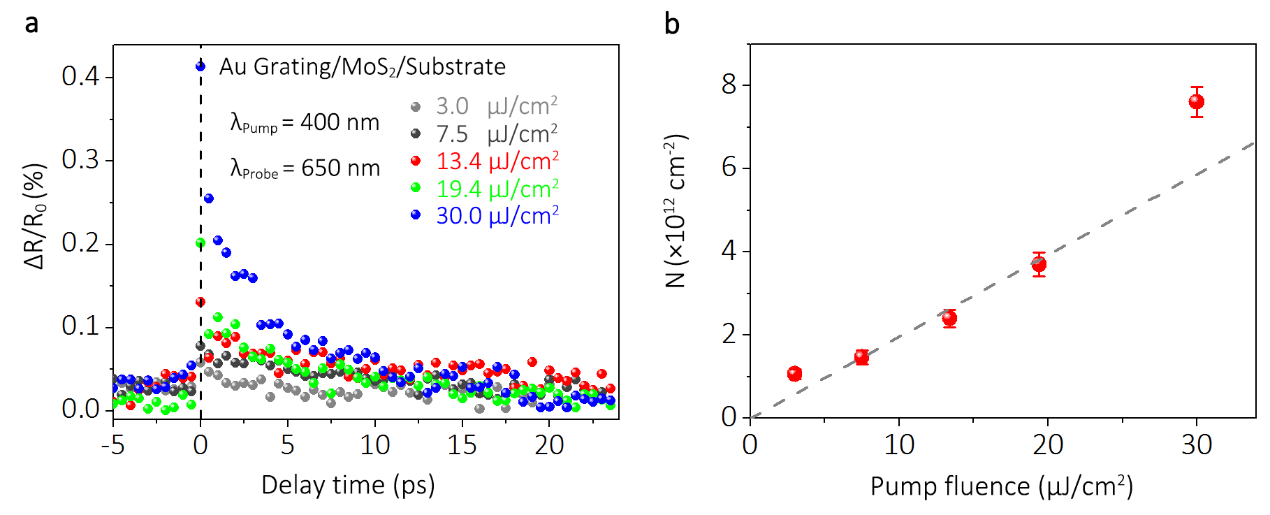


**Figure S6.** Ultrafast pump-probe spectra of the heterostructure pumped at 400 nm. (a) Differential reflection spectra for the sample with 700 nm period as the pump fluence increases. (b) Excited electron densities at different pump fluences. The dashed line represents directly calculated electron densities according to the absorbance of MoS_2_ monolayer, while solid points correspond densities derived from peak amplitudes of ΔR/R_0_(t) with the linear relationship.


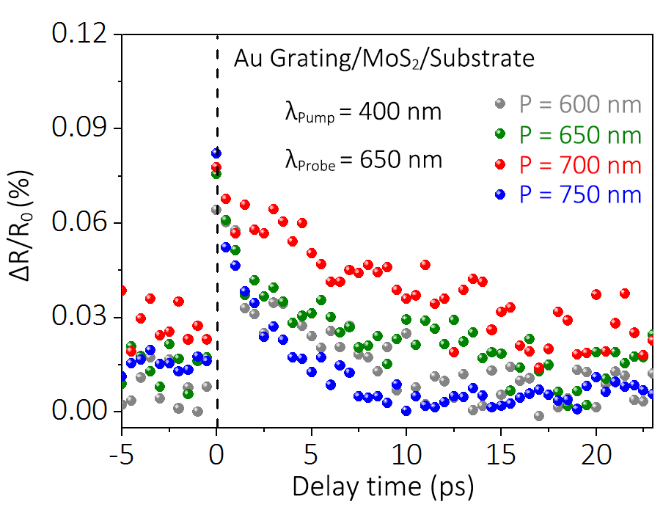


**Figure S7.** Differential reflection spectra for four different periods. The pump wavelength was 400 nm with fluence 7.5 μJ/cm^2^. In contrast to the results pumped at 780 nm, peak amplitudes are not greatly varied with the grating period.


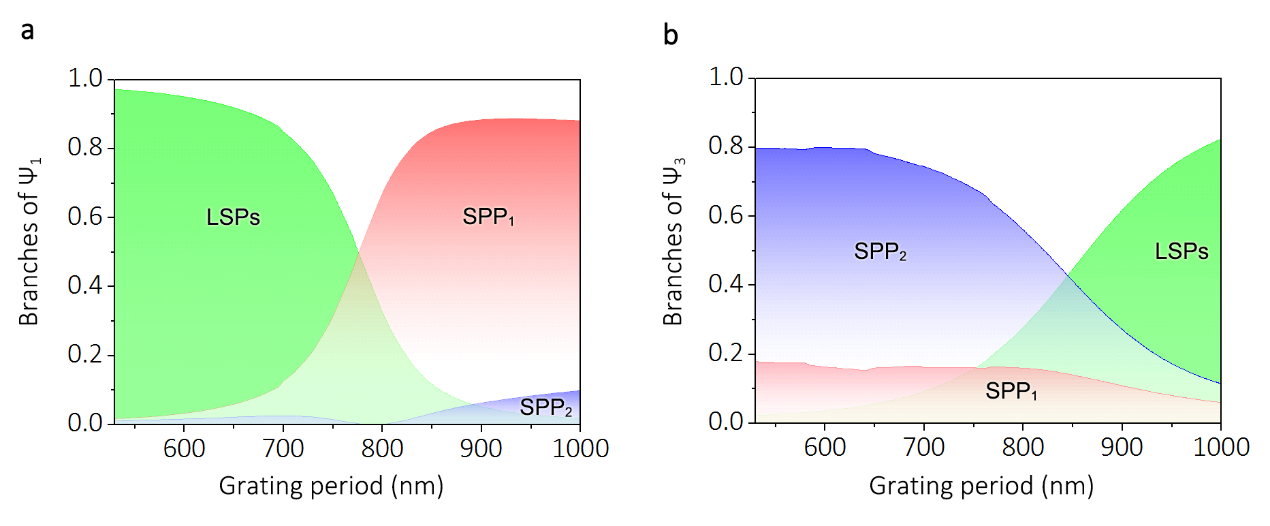


**Figure S8.** Branches of Ψ_1_ and Ψ_3_ as a function of grating period. Green, red and blue areas represent branches of uncoupled LSPs, SPP_1_ and SPP_2_ modes. For the period of 700 nm, LSPs contribute 85% (10%) to Ψ_1_ (Ψ_3_). Consistent with electromagnetic distributions in Figure 2c and Figure S3, Ψ_1_ is dominated by LSPs, while Ψ_3_ is mainly comprised of SPP_2_.

**Reference**

1 Kitson SC, Barnes WL, Sambles JR. Full Photonic Band Gap for Surface Modes in the Visible. *Phys Rev Lett* 1996; **77**: 2670-2673.

2 Kitson SC, Barnes WL, Bradberry GW, Sambles JR. Surface profile dependence of surface plasmon band gaps on metallic gratings. *J Appl Phys* 1996; **79**: 7383-7385.

3 Barnes WL, Preist TW, Kitson SC, Sambles JR, Cotter NPK *et al.* Photonic gaps in the dispersion of surface plasmons on gratings. *Phys Rev B* 1995; **51**: 11164-11167.

4 Barnes WL, Preist TW, Kitson SC, Sambles JR. Physical origin of photonic energy gaps in the propagation of surface plasmons on gratings. *Phys Rev B* 1996; **54**: 6227-6244.

5 Hong X, Kim J, Shi S-F, Zhang Y, Jin C *et al.* Ultrafast charge transfer in atomically thin MoS_2_/WS_2_ heterostructures. *Nat Nanotechnol* 2014; **9**: 682-686.

6 Zhu H, Wang J, Gong Z, Kim YD, Hone J *et al.* Interfacial Charge Transfer Circumventing Momentum Mismatch at Two-Dimensional van der Waals Heterojunctions. *Nano Lett* 2017; **17**: 3591-3598.

7 Peng B, Yu G, Zhao Y, Xu Q, Xing G *et al.* Achieving Ultrafast Hole Transfer at the Monolayer MoS2 and CH3NH3PbI3 Perovskite Interface by Defect Engineering. *ACS Nano* 2016; **10**: 6383-6391.

8 Liu HL, Shen CC, Su SH, Hsu CL, Li MY *et al.* Optical properties of monolayer transition metal dichalcogenides probed by spectroscopic ellipsometry. *Appl Phys Lett* 2014; **105**: 201905.

9 Li Y, Chernikov A, Zhang X, Rigosi A, Hill HM *et al.* Measurement of the optical dielectric function of monolayer transition-metal dichalcogenides: MoS_2_, MoSe_2_, WS_2_, and WSe_2_. *Phys Rev B* 2014; **90**: 205422.

10 Sun D, Rao Y, Reider GA, Chen G, You Y *et al.* Observation of Rapid Exciton–Exciton Annihilation in Monolayer Molybdenum Disulfide. *Nano Lett* 2014; **14**: 5625-5629.

11 Wang R, Ruzicka BA, Kumar N, Bellus MZ, Chiu H-Y *et al.* Ultrafast and spatially resolved studies of charge carriers in atomically thin molybdenum disulfide. *Phys Rev B* 2012; **86**: 045406.
